# “TORNADO” – Theranostic One-Step RNA Detector; microfluidic disc for the direct detection of microRNA-134 in plasma and cerebrospinal fluid

**DOI:** 10.1038/s41598-017-01947-2

**Published:** 2017-05-11

**Authors:** Hazel McArdle, Eva M. Jimenez-Mateos, Rana Raoof, Eadaoin Carthy, David Boyle, Hany ElNaggar, Norman Delanty, Hajo Hamer, Muejgdan Dogan, Tessa Huchtemann, Peter Kӧrtvelyessy, Felix Rosenow, Robert J. Forster, David C. Henshall, Elaine Spain

**Affiliations:** 1 School of Chemical Sciences, National Centre for Sensor Research, Dublin City University, Dublin 9, Ireland; 2 Department of Physiology and Medical Physics, Royal College of Surgeons in Ireland, Dublin 2, Ireland; 3 Neurological Services, Beaumont Hospital, Dublin 9, Ireland; 4 University Hospital Erlangen, Schwabachanlage 6, 91054 Erlangen, Germany; 5 Department of Neurology, University hospital Magdeburg, Leipziger Strasse 44, 39120 Magdeburg, Germany; 6 German Center for Neurodegenerative Diseases (DZNE), Magdeburg, Leipziger Strasse 44, 39120 Magdeburg, Germany; 7 Epilepsy Center Hessen, Department of Neurology, Baldingerstr, 35043 Marburg Germany; 8 Epilepsy Center Frankfurt Rhine-Main, Neurocenter, Goethe-University, Schleusenweg 2-16, Haus 95, 60528 Frankfurt a.M., Germany

## Abstract

Diagnosis of seizure disorders such as epilepsy currently relies on clinical examination and electroencephalogram recordings and is associated with substantial mis-diagnosis. The miRNA, miR-134 (MIR134 in humans), has been found to be elevated in brain tissue after experimental status epilepticus and in human epilepsy cells and their detection in biofluids may serve as unique biomarkers. miRNAs from ***unprocessed human plasma*** and ***human cerebrospinal fluid*** samples were used in a novel electrochemical detection based on electrocatalytic platinum nanoparticles inside a centrifugal microfluidic device where the sandwich assay is formed using an event triggered release system, suitable for the rapid point-of-care detection of low abundance biomarkers of disease. The device has the advantage of controlling the rotation speed of the centrifugal device to pump nanoliter volumes of fluid at a set time and manipulate the transfer of liquids within the device. The centrifugal platform improves reaction rates and yields by proposing efficient mixing strategies to overcome diffusion-limited processes and improve mass transport rates, resulting in reduced hybridization times with a limit of detection of 1 pM target concentration. Plasma and cerebrospinal fluid samples (unprocessed) from patients with epilepsy or who experienced status epilepticus were tested and the catalytic response obtained was in range of the calibration plot. This study demonstrates a rapid and simple detection for epilepsy biomarkers in biofluid.

## Introduction

Epilepsy is a common neurological disease characterised by an enduring predisposition to recurrent seizures^1, 2^. Seizures can also occur in patients without epilepsy as a result of a systemic disturbance (e.g. infection). Prolonged seizures, termed status epilepticus, are a neurological emergency and non-convulsive SE is extremely hard to diagnosis due to the not specific EEG pattern in NCSE meaning they are frequently misdiagnosed. Diagnosis of both epilepsy and status epilepticus is sometimes challenging and often relies heavily on clinical examination and history alone. The primary tool used for diagnosis of seizure disorders is the electroencephalogram (EEG). While invaluable, EEG is costly and technically demanding^3^. Moreover, many patients with epilepsy have a normal EEG recording while patients without epilepsy can have apparently abnormal EEG findings. As a result there are high mis-diagnosis rates for epilepsy and status epilepticus. Identifying a molecular biomarker of seizures in a biofluid such as blood, urine or cerebrospinal fluid (CSF) would vastly improve the diagnosis, prognosis, care and treatment of these patients^4, 5^. Many efforts have been focussed on areas such as antibodies to neuronal antigens, infectious markers, inflammatory markers, white blood cells and associated cell adhesion molecules paediatric syndromes, and treatment-related biomarkers; however these have been largely unsuccessful^6^.

A promising class of biomarkers for epilepsy are microRNA^7^. MicroRNAs are an important class of small noncoding RNA which function to negatively regulate protein levels in cells by post-transcriptional interference in gene expression. A number of miRNA have been found to be selectively enriched in specific brain cell types, including miR-134, which influences the strength of inter-neuronal signalling by targeting proteins that shape microstructures called dendrites^8, 9^. Recent studies reported upregulation of miR-134 in rodent models of status epilepticus and human epilepsy^10^, and have shown that inhibiting miR-134 had long-lasting seizure-suppressive effects in mice^11^. Recent work has shown that levels of a number of miRNAs are altered in blood following seizures in rodents^12, 13^, and in epilepsy patients^14, 15^. The detection of one or more brain-specific miRNA in biofluids such as plasma or cerebrospinal fluid (CSF) may support diagnosis, predict seizures or guide treatment decisions for patients with epilepsy or status epilepticus^14^.

The ability to detect biomarkers such as brain-derived miRNAs rapidly and at ultralow concentrations is a major focus of sensor research. Various techniques are used for the detection of miRNA, including fluorescence^16^, chemiluminescence^17^, gravimetric^18^, surface plasmon resonance^19^, and electrochemistry^20^. Electrochemical biosensors have the advantage of being cheap, user friendly, sensitive, and selective, and therefore is a promising technique for use in point-of-care devices^21, 22^. One of the biggest challenges for the development of biosensors is the limit of detection, as the miRNA concentration could be as low at attomolar to femtomolar in biological samples^23, 24^. In the case of miR-134, biofluid levels may be exceptionally low since it is expressed in mainly a subset of brain cells. Thus, a 100 µl sample of patient’s blood may contain less than 10,000 copies of the biomarker. Also, these molecules must be detected in the presence of proteins and other nucleic acids that could interfere with the detection. Previous work^25^ has shown that miR-134 can be detected at low levels using platinum nanoparticles (PtNPs) that are region-selectively decorated with probe strand nucleic acids complementary to miR-134 target in serum samples from epilepsy patients. The PtNPs are brought to the surface of the electrode via miRNA hybridisation complementary to the target. The target concentration was detected by the current associated with the reduction of hydrogen peroxide at the electrode surface and a limit of detection at a sub-attomolar level was achieved. The reported system required extraction of the miRNA from the biofluid before electrochemical detection was carried out. These samples showed highly linear correlation in miR-134 measurement when compared to results obtained from Taqman-based PCR^25^. The previously described assay required large volumes of the initial biofluid samples to obtain enough isolated miRNA target for hybridization. The choice of a matrix for the detection of miRNAs can have a direct impact on the expression profiles of these novel biomarkers. miRNAs can be extracted with standard protocols such as TRIzol-based reagents or with commercial kits favoring small RNA enrichment. While, different approaches are reported to be suitable, discrepancies have been noted when comparing different methods of extraction^26^. Since blood is considered to be a matrix with low levels of miRNAs, this issue needs to be considered seriously. Here we demonstrate the detection of miRNA in an unprocessed biofluid samples as a routine detection strategy in a clinical setting, in a microfluidic device.

A Theranostic One-Step RNA Detector (“TORNADO”) is described for the direct detection of microRNA-134 in plasma and cerebrospinal fluid from patients who experienced seizures. Centrifugal platforms for bioanalytical assays have been investigated for more than 40 years^27, 28^. Centrifugal microfluidic platforms offer many advantages over chip-based microfluidic systems such as, minimal instrumentation without any pumps, inexpensive materials that can be mass produced and it is not dependant on physiochemical properties such as pH, ionic strength or chemical composition so many different fluid samples can be used^29^. This centrifugal microfluidic platform, in particular not only has the advantage of a directed flow of biofluids providing high uniformity and reproducibility, but also decreases sample volumes significantly. This small volume reduces the sample quantity needed for appropriate concentrations and can thus be used with significantly smaller amounts of patient sample.

Here we report the use of electrocatalytic platinum nanoparticles are functionalised with probe strand miRNA that are complementary to a particular region of the target, miR-134, and are used to detect this target strand without PCR amplification of the target. Thiol terminated probe strand miRNA are immobilised onto spherical platinum nanoparticles and these are pre-loaded into a microfluidic disc, along with the target miRNA, miR-134. Capture miRNA that is complementary to part of the target miRNA are immobilised via thiol bonding to a bare gold electrode, and is assembled into a microfluidic disc. Using a triggering system, and by controlling the force at which the disc is spinning on an experimental spin stand, the pre-loaded target miRNA and probe-functionalised platinum nanoparticles can be released at specific times, for a specific duration, in order to expose the capture strand functionalised to each step, to attach the electrocatalytic particles to the electrode surface, via the target miRNA hybridisation. When the electrode is fully functionalised, electrochemical detection is carried out on the disc, by connecting the external contact of the electrode to a potentiostat.

## Experimental

### Materials

Denhardt’s Hybridisation solution (≥99.5%) for miRNA strand assemble was used as received from Sigma Aldrich. Platinum nanoparticles (50–70 nm) were purchased from Strem Chemicals. All aqueous solutions were prepared using RNase free water. The oligonucleotides were purchased from Eurogentec and their purity was >98%. The base sequences are as follows:

Capture: 5′-ACC-AGU-CAC-A-3′-SH;

Target (miR-134): 5′-UGU-GAC-UGG-UUG-ACC-AGA-GGG-G-3′;

1-base mismatch (miR-758): 5′-UGU-GAC-UGG-UUG-ACC-AGA-GAG-G-3′;

Neuroblastoma: 5′-UAA-CAG-UCU-ACA-GCC-AUG-GUC-G-3′

S. aureus: 5′-AAG-CCG-GTG-GAG-TAA-CCT-TTT-AGG-AGC-3′

MRSA: 5′-TAA-CAG-TCT-ACA-GCC-ATG-GTC-G-3′Probe: SH-5′-CCC-CUC-UGG-U-3′.

### Instrumentation

**Figure 1:**
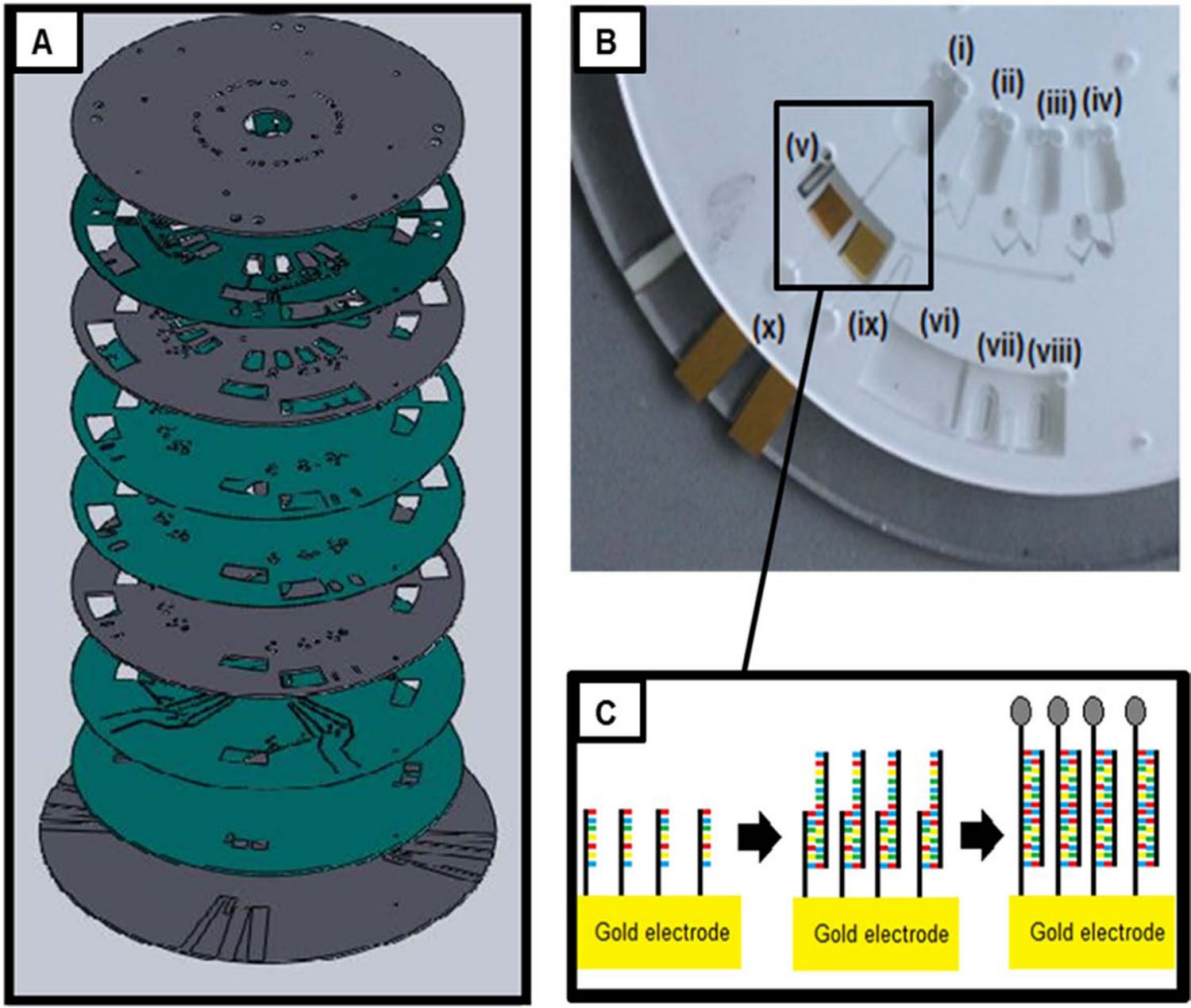
(**A**) Detailed view of disc assembly. The grey layers are PMMA, the green layers are PSA. (**B**) Image of fully assembled disc with integrated electrodes (cropped to view one section). Chamber (i–iv) are preloaded with (i) target miRNA strands, (ii) PBS wash step, (iii) PtNP labelled probe miRNA strands, (iv) PBS wash step/electrolyte. Chamber (v) is the electrode chamber with ITO reference, gold working electrode and gold counter electrode. Chamber (vi) is the waste chamber, with an overflow system implemented at (vii) and (vii); these contain DF tabs. (ix) shows the siphon between the electrode chamber and the waste chamber. Labelled at (x) are the vents for the electrode chamber. (**C**) Hybridisation of miRNA strands on surface of working electrode inside chamber (v).

The amperometric measurements for miRNA detection were performed using a CH Instruments, Model 760D electrochemical workstation. A three-electrode electrochemical cell was used at a temperature of 22 ± 2 °C. This was inside the electrode chamber of the microfluidic device (Fig. 1). The working electrode and the counter electrode were a gold coated silicon wafer (Amsbio) cut into 0.5 cm wide slides. The slides were immersed in ethanol for 5 minutes and rinsed with Milli-Q water prior to use. An ITO slide acted as the reference electrode.

## Methods

### All methods were carried out in accordance with the relevant guidelines and regulations

#### Disc design and assembly

The microfluidic disc (Fig. 1A) was assembled from 4 layers of poly(methyl methacrylate) (PMMA) and 5 layers of pressure sensitive adhesive (PSA). Larger voids such as reservoirs and vents were machined in PMMA layers using a CO_2_ laser cutter. PMMA layers were 0.5 mm, 1.5 mm or 2 mm thick. Small features such as microchannels and lower channels were created from voids cut out in PSA using a knife-cutter. Layer 1 (1.5 mm PMMA) consisted of the vents. Layer 2 (PSA) consisted of the microchannels for liquid transport. Layer 3 (1.5 mm PMMA) provided large reservoirs. Layer 4 (PSA) was a cover layer which sandwiches the dissolvable film (DF) tabs in place. Layer 5 (PSA) was a support layer for the DF tabs. Layer 6 (0.5 mm PMMA) consisted on the midlayer containing through holes. Layer 7 (PSA) features the lower channels for fluid flow. Layer 8 (PSA) was the electrode cover. Layer 9 (2 mm PMMA) consisted of the rastered base which held the electrodes.

The dissolvable film (DF) tabs were made of polyvinyl alcohol (PVA), and attached to double-sided PSA to create adhesive tabs^30^. Circular shaped and slot shaped tabs were both used; the circular tabs were used for the load film (LF) at the sample chambers; the slot-shaped tabs were used for the control film (CF) at the waste chambers.

The disc was designed to allow the pre-loading of the chambers labelled (i–iv) in Fig. 1B; these were pre-loaded with: (i) target miRNA/sample; (ii) DPBS (wash step); (iii) probe miRNA functionalised platinum nanoparticles; (iv) DPBS. Chamber (v) was the electrode chamber. The chambers labelled (vi–viii) were the waste chambers. A siphon was used between the electrode chamber and the waste chamber, shown at label (ix). This was used to prevent the fluid from flowing from the electrode chamber to the waste chamber prematurely, to allow sufficient incubation time, as when the disc is spun on the spin stand at a high spin rate, the fluid cannot rise above the curve in the siphon channel as the centrifugal force is stronger than the capillary force^31, 32^. A venting system was implemented (shown at label (x)) below the electrode chamber to allow the release of the gases produced during the electrocatalytic reduction of hydrogen peroxide.

The disc was mounted on an experimental spin stand^33–35^. The discs were spun on a computer controlled motor. A stroboscopic light source, a sensitive, short exposure time camera and the motor are synchronized using custom electronics and visualise the hydrodynamics on the rotating disc. The discs were tested at varying rates of rotation, ranging from 1 Hertz to 35 Hertz, depending on the stage of testing. This utilised the DF tabs and event-triggering release of the chambers, and is explained further in the results section.

#### Human plasma and CSF samples

Studies were approved by the Research Ethics Committee of the Royal College of Surgeons in Ireland (RED #859) and by the Ethics (Medical Research) Committee of Beaumont Hospital, Dublin. Informed written consent was obtained from all patients and volunteers. Blood was collected by venupuncture into K2-EDTA tube, 10 ml, BD cat. No. 367525, gently inverted 8–10 times, and processed to obtain plasma within one hour. Plasma was prepared by centrifuging the tubes at 1300 × g, for 10 mins at 4 °C. A second centrifugation step was performed at 1940 × g for 10 min at 4 °C to further reduced cellular contamination^36^. After centrifugation, samples were decanted for storage in a cryo-tube (Greiner Bio-one) and frozen at −80 °C.

Cerebrospinal fluid (CSF) samples were collected from patients using a standard lumber puncture procedure from a sitting position or lying on their side. CSF was centrifuged within one hour of collection at 300 × g for 10 mins at 4 °C to remove contamination or cellular debris, the supernatant was collected and stored at −80 °C until use. CSF samples were collected from two different centres during clinical workup: the University of Magdeburg, and The Friedrich-Alexander-University Erlangen-Nurnberg. Consent was obtained according to the Declaration of Helsinki and ethical approval was obtained from the local medical ethics committees at each center. This included informed written consent given by patients or their legal representatives if the patients were obtunded.

#### Patients

Plasma was obtained from two (2) healthy volunteers (female, 38; male, 25) and three (3) temporal lobe epilepsy patients attending the video EEG monitoring unit at Beaumont hospital for epilepsy diagnosis. CSF samples were obtained from three (3) patients one with refractory TLE and two with SE due to causes other than epilepsy. Patient data are presented in Table 1. All patients were on medication at the time of the study.

**Table 1:** Summary of patient and healthy volunteer data.

Patient	Age	Sex	Sample	Disease	Comments
A	38	F	plasma	n/a	n/a
B	25	M	plasma	n/a	n/a
C	42	M	plasma	TLE	automotor and GTC seizures
D	19	M	plasma	TLE	automotor and GTC seizures
E	64	F	plasma	TLE	automotor and GTC seizures
F	56	F	CSF	TLE	automotor and GTC seizures
G	91	M	CSF	SE	focal status with motor symptoms over 6 hours
H	60	M	CSF	SE	focal status with motor symptoms over 6 hours

Key: F, female; M, male; CSF, cerebrospinal fluid; TLE, temporal lobe epilepsy; SE, status epilepticus; GTC, generalised tonic clonic.

#### miRNA hybridisation; fabrication of sandwich assay in microfluidic disc and detection of the miRNA target

By utilising this sequential, event-triggered release of each chamber on this disc, the miRNA strands can hybridise to form the sandwich assay shown in Fig. 1C. The gold slide working electrode was pre-functionalised with capture miRNA by immersing the electrode in a 1 µM solution of the capture oligo strand dissolved in Denhardt’s buffer for 30 minutes. The capture strand is complementary in part to the target miRNA, miR-134. This was then rinsed with RNase free water to remove any loosely bound oligonucleotides and dried under nitrogen before being assembled in the microfluidic device as explained above. The disc was then spun on the experimental spin stand; when the first chamber containing differing concentrations of the target was released, it was left in the electrode chamber for 30 minutes to incubate. This allowed the target to hybridise to the complementary capture miRNA on the electrode surface. The electrode was rinsed by release of the second sample chamber, containing DPBS. The third chamber contained 1 µM probe miRNA functionalised platinum nanoparticles (50–70 nm); this probe strand was complementary to the non-hybridised part of the target miRNA. When this was released into the electrode chamber, it was left for 30 minutes to incubate to allow the probe and target to hybridise together. The sandwich assay shown in Fig. 1C was then formed on the electrode. When chamber 4, (containing DPBS) was released, this remaining in the electrode chamber for electrochemical analysis. The external elements of the electrode were connected to a potentiostat.

Following assembly of the capture-target-nanoparticle labelled probe miRNA sequence, the current was measured at −0.25 V after equilibrium for 10 minutes. Sufficient hydrogen peroxide was then added to give a final concentration of 20 µM and the current was measured at −0.25 V after equilibrium for 20 minutes. The analytical response is taken as the difference is current, Δi, measured before and after peroxide addition.

### Ethical approval

Studies were approved by the Research Ethics Committee of the Royal College of Surgeons in Ireland (REC #859) and by the Ethics (Medical Research) Committee of Beaumont Hospital, Dublin. Informed written consent was obtained from all patients and volunteers. CSF samples were collected from two different centres during clinical workup: the University of Magdeburg, and The Friedrich-Alexander-University Erlangen-Nurnberg. Consent was obtained according to the Declaration of Helsinki and ethical approval was obtained from the local medical ethics committees at each center. This included informed written consent given by patients or their legal representatives if the patients were obtunded.

## Results and Discussion

### Step-wise functionalisation and hybridisation of miRNA using triggering system of microfluidic device

The valving technology implemented here uses the arrival of a liquid at one location to prompt the release of another liquid at another, distant location on the disc by a connecting pneumatic channel. This enables the multi-step fluid handling sequence that is required to make the sandwich assay used for the nucleic acid detection. An overflow system was implemented in the waste chambers also; this means that only when the second sample chamber (labelled (ii) in Fig. 1) has emptied into the waste chamber, it then flows into the overflow part of the waste chamber (vii), which contains a control film (CF). When this CF gets wet, it vents the pneumatic -channel (Lower channels, Layer 7) permitting the sample chamber (iii) to advance, and wet and dissolve the load film (LF). This liquid could then flow into the electrode chamber. When this pneumatic chamber (iii) was vented, the liquid can only flow into the electrode chamber and not back through the venting channel into the waste; this was achieved through a physical barrier by extending the microchannels linking the CF and LF (lower channels, layer 7) radially inward of the fluid in the sample chamber; this forces the fluid into the electrode chamber.

**Figure 2:**
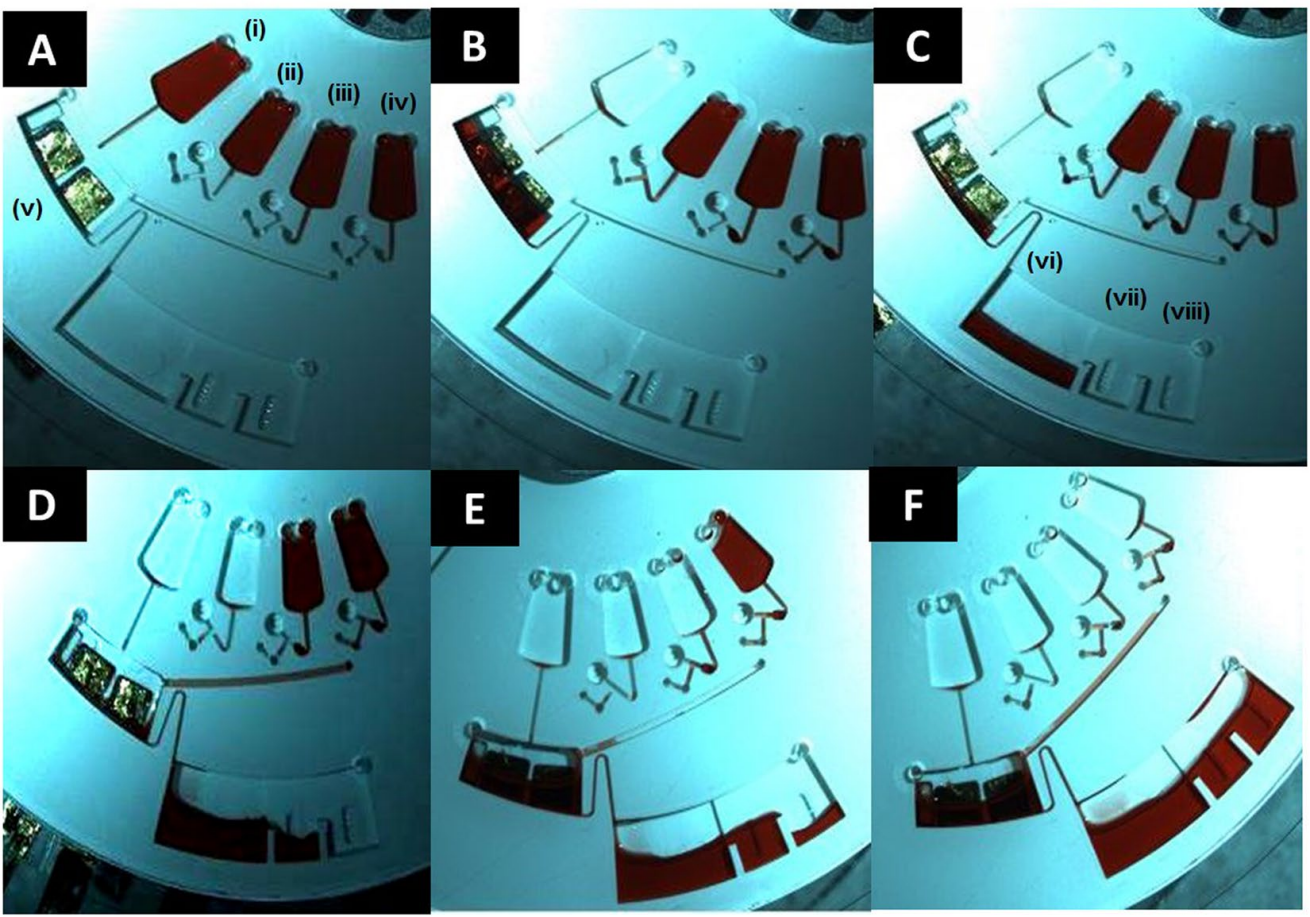
Stepwise display of the triggering system implemented to allow complete functionalisation of the working electrode with the sandwich assay in the incubation chamber of the microfluidic device. Each image is cropped to show just one section of the disc. Red food dye is preloaded into the chambers instead of samples for visualisation purposes only. Chambers (i–iv) are the preloaded sample chambers. Chamber (v) is the electrode chamber. Chamber (vi) is the waste chamber, with (vii) and (viii) as the overflow of the waste.

This triggering system is shown in Fig. 2, using red food dye instead of the sample for clarity.

**Image A:** The sample chambers (i–iv) were preloaded as described above.

**Image B:** A spin rate of 15 Hz was applied. The forced the contents of chamber (i) into the electrode chamber (v). This was left to incubate for 30 min, so the target miRNA could hybridise to the capture miRNA immobilised on the surface of the working electrode.

The siphon between the electrode chamber (v) and the waste chamber (vi) (shown as (ix) on Fig. 5.3) prevents the flow of the sample into the waste. After the 30 min incubation time, the spin rate was slowed down to 1 Hz; this allowed the siphon to prime.

**Figure 5:**
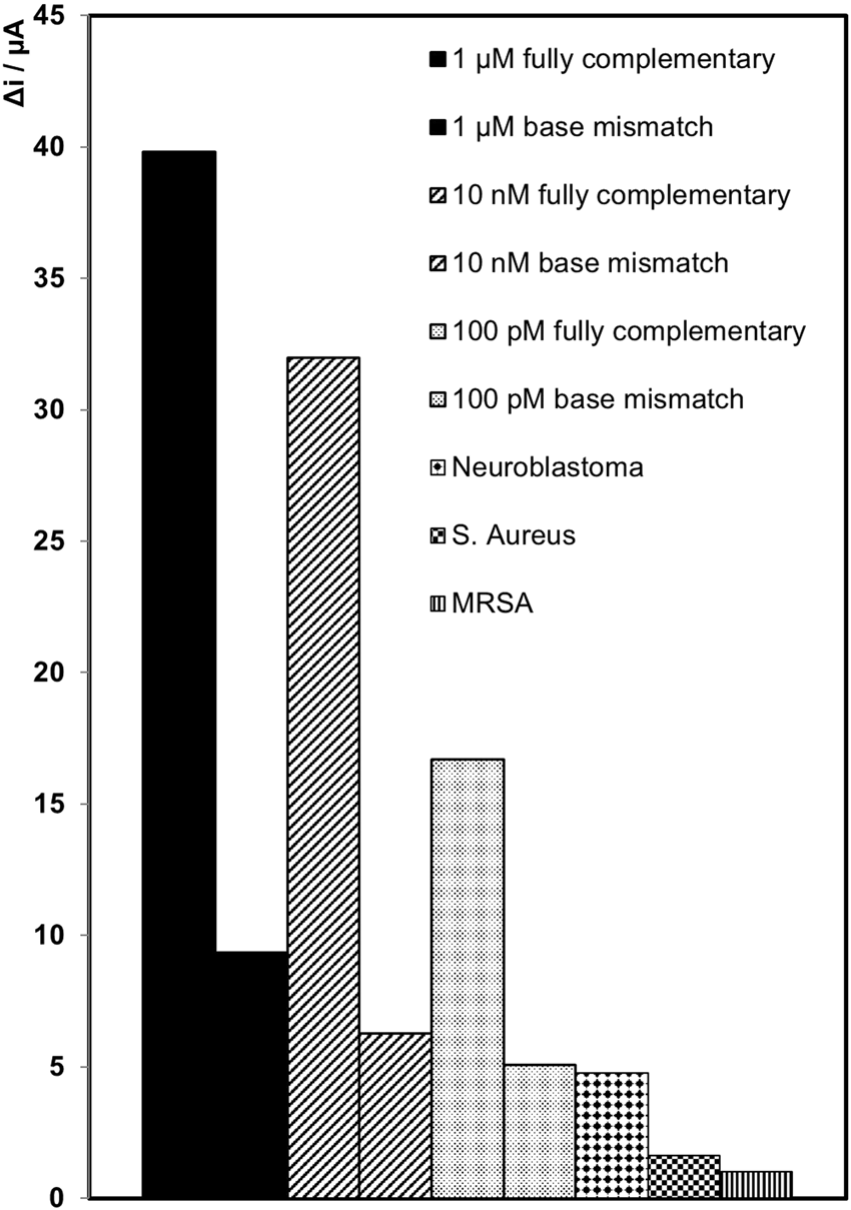
Comparison of the change in current for the fully complementary target strand (miR-134), the one base mismatch (miR-758) target strand and fully non-complimentary neuroblastoma (black diamonds), S. aureus (black squares), and MRSA (black vertical lines) nucleic acid strands. Three different target concentrations were used for miR-758; 1 µM (solid black), 10 nM (black lines) and 100 pM (black dots). Concentration of the miR-132-3p, SA, and MRSA nucleic acid target strands are 1 µM. Concentration of capture and probe miRNA strands are 1 µM, where the probe miRNA strand is labelled with electrocatalytic PtNPs. Concentration of H_2_O_2_ added is 20 µM. Potential applied is −0.25 V in 1 mM DPBS.

**Image C:** Once the siphon was primed, the spin rate was increased to 10 Hz, to force the sample into the waste chamber.

**Image D:** Once the entire sample was in the waste chamber, the spin rate was increased to 30 Hz. This high spin rate released the sample chamber (ii) which flowed into the electrode chamber. This is a wash step, so the fluid can flow over the electrode and through the siphon into the waste chamber.

As an overflow system was implemented here, the fluid overflows from the waste chamber (vi) into the overflow chamber (vii). This wetted the DF tab in the chamber, releasing the pressure in the lower channel, allowing the DF tab at chamber (iii) to become wet.

**Image E:** As the DF tab of chamber (iii) was wetted, the spin rate was increased to 35 Hz to allow this chamber to empty into the electrode chamber. This chamber contained the probe functionalised platinum nanoparticles and was left for 30 min to incubate, to allow the probe to hybridise to the target miRNA on the surface of the working electrode.

When the incubation time was complete, the spin rate was slowed down once again to prime the siphon and allow flow into the waste chamber. Another overflow system was implemented here; as the volume increased, the fluid flowed into waste chamber (viii) and wetted the DF tab in this chamber, releasing the pressure in the lower channels, so the DF tab at sample chamber (iv) can be wetted also.

**Image F:** The spin rate was increased to 35 Hz once again, to allow the release of the liquid in chamber (iv) into the electrode chamber, where it remained for electrochemical testing.

The disc was removed from the spin stand and the external elements of the electrodes were connected to a potentiostat for testing.

### Electrocatalytic detection of miR-134 in clean buffer

Platinum nanoparticles are well known to be highly electrocatalytic for the reduction of hydrogen peroxide^37^. Platinum nanoparticles are confined on the gold electrode by complementary miRNA hybridisation. These platinum nanoparticles are capable of electrocatalysing the reduction of hydrogen peroxide, generating a current that is directly proportional to the number of nanoparticles on the surface of the electrode. The number of nanoparticles on the surface of the electrode depends directly on the concentration of target miRNA. A fixed potential of −0.25 V was applied to the working electrode and the difference in current in the absence of H_2_O_2_ and after the addition of 20 µM H_2_O_2_ was measured. The peroxide was injected into the electrode chamber on the microfluidic disc, which contains 1 mM DPBS.

**Figure 3:**
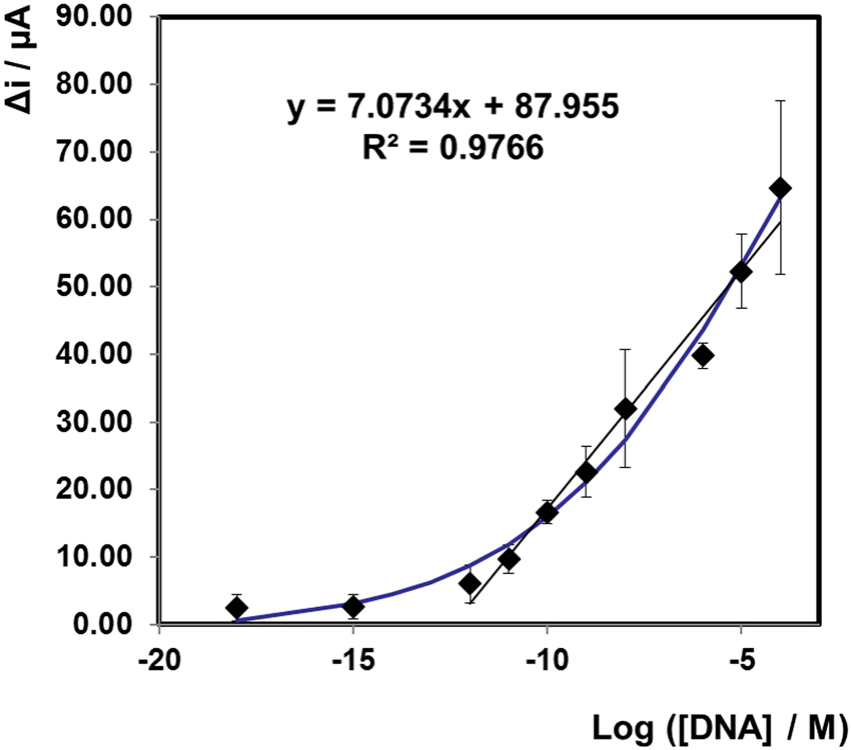
Dependence of the difference in current before and after the addition of H_2_O_2_ on log[miRNA] on a gold slide electrode following hybridisation with probe miRNA that is labelled with PtNPs. The applied potential is −0.25 V in 1 mM DPBS. Δi represents the difference in current before and after addition of 20 µM H_2_O_2_. Error bars are from an n = 3 study and range from 4–13%. The calibration plot is fit with a 4 parameter logistic function (blue line).

Figure 3 shows the dependence of the Δi on log[miRNA] using varying concentrations of miR-134 in clean buffer that are labelled with platinum nanoparticles. For this full sandwich assay, an acceptably linear response (R^2^ = 0.9766) is observed for concentrations ranging from 1 µM to 1 pM, with high sensitivity and a wide dynamic range over six orders of magnitude. The wide dynamic range is due to the area of occupation of an individual nanoparticle is small and even for high concentrations, sufficient area is available on the electrode. The dissociation constant for the target to capture hybridisation is 6.8 × 10^11^ M and the dissociation constant for the probe to target hybridisation is 8.47 × 10^14^ M, showing that the equilibrium is exclusively on the hybridisation side. The shape of the curve is typical of an S-shaped calibration curve, i.e. it will have an upper and a lower detection limit and the calibration plot was fit with a 4-parameter logistic function, as shown in Fig. 3 (blue line). Limit of detection (LOD) is defined as the lowest analyte concentration likely to be reliable from the blank and is determined by utilising the measured Limit of the Blank (LOB), and test replicates of a sample containing low concentrations of the target;

$${\rm{LOD}}={\rm{mean}}\,{\rm{of}}\,{\rm{blank}}+3{\rm{\sigma }}$$

For this system the LOD is calculated to be 1 × 10^−12^ M (1 pM). This is the point on the curve where the current no longer depends on the concentration. The LOD of detecting miR-134 within a centrifugal device is considerably higher than our previously reported bench top experiments of extracted miRNA serum samples (atto molar)^25^. The geometry of the device has the advantage of controlling the rotation speed of the centrifugal device to pump nanoliter volumes of fluid at a set time and manipulate the transfer of liquids within the device. Due to the shallow architecture of the microchannels, the probability of collision between the miRNA hybridised on the electrode surface and the target (miR-134) is increased by the much shorter diffusion distance, thereby accelerating the hybridisation kinetics. The centrifugal platform therefore improves reaction rates and yields by proposing efficient mixing strategies to overcome diffusion-limited processes and improve mass transport rates, resulting in reduced hybridisation times and increased LOD when compared to bench top experiments.

**Figure 4:**
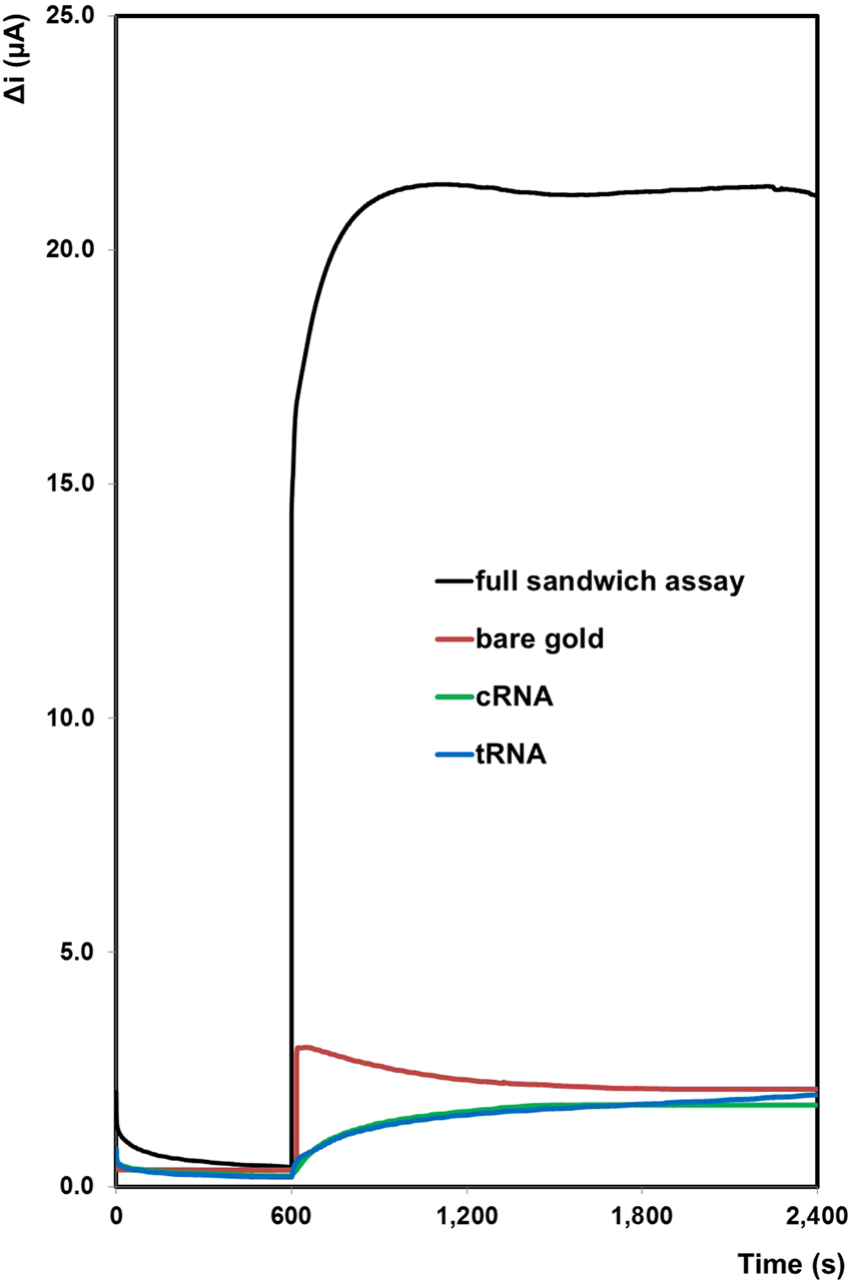
Amperometric i-t curves for a bare gold electrode (red line), a gold electrode functionalised with 1 µM capture miRNA (cRNA, green line), a gold electrode functionalised with 1 µM capture miRNA and hybridised to 1 µM target miRNA (tRNA, blue line), and a gold electrode functionalised with capture, 1 nM target and probe miRNA labelled with platinum nanoparticles (full sandwich assay, black line). Potential applied is −0.25 V in 1 mM DPBS. The difference in current before and after the addition of 20 µM H_2_O_2_ is displayed.

In a clinical environment, the LOD is well within the range needed to pick up even a low-abundance, brain-specific miR like miR-134, especially from unprocessed biofluids. This LOD compares favorably with other centrifugal platforms that have been developed for nucleic acid detection. However, many of these require the nucleic acid to be amplified by methods such as PCR^38, 39^, LAMP^40^, and RCA^41^. These have some disadvantages though, as they require thermal cycling, can have large background noise, complicated primers required for amplification, and can sometimes give false positives^1^. TORNADO has the advantage of simply detecting miRNA from a neat unprocessed biofluid sample in an integrated device without the requirement to reverse transcribe, generate cDNA and run PCR for amplification of the DNA, leading to a much simpler and quicker detection method for clinical utility. This device has the advantage of quickly and simply detecting miRNA (1.5 hours sample-to-answer) from a neat unprocessed biofluid sample in an integrated device without relying on amplification or purification of the target, making it attractive for clinical setting. Figure 4 shows the current change before and after the addition of 20 µM H_2_O_2_ for each step of the hybridisation process inside the microfluidic device. As expected, when the target is not labelled with the platinum nanoparticles there is no significant current produced. The low currents that are observed are due to the background current of the underlying electrode, which is shown to be very low. This result is significant as it is the signal-to-noise ratio that dictates the limit of detection that can be achieved and also reflects the poor electrocatalytic properties of a miRNA modified gold electrode, and demonstrates the need for a platinum nanoparticle label. The comparison of these small currents to the relative large current for the fully complementary, labelled samples suggests that the heterogeneous electron transfer from the platinum nanoparticles, through the miRNA linker to the underlying electrode is relatively facile. Also, because nanoparticles are used as electrocatalytic labels, the rate of mass transport of H_2_O_2_ is enhanced in comparison to a planar electrode, due to radial diffusion.

The selectivity of the sensor was also investigated using target miRNA sequence that contained a single mismatch and other, non-complementary nucleic acid strands. The structure of miR-758 is the most similar to miR-134; this contains a sequence of 12 bases that are the same as that found in miR-134. Fully non-complementary nucleic acid strands were also used; miR-132-3p, which is associated with the childhood cancer Neuroblastoma, a DNA sequence associated with Staphylococcus aureus and a DNA strand that is associated with methicillin resistant staphylococcus aureus. Figure 5 shows a comparison of the fully complementary target miRNA strand with the 1 base mismatch, miR-758 for three different target strand concentrations, and the non-complimentary nucleic acid strands. For each concentration of the 1 base mismatch, the current generated is 70–80% lower than that for the fully complementary target sequence. For the non-complementary nucleic acid strands, the current generated is 88–98% lower than that for the fully complementary target miRNA sequence This indicates that the current is dominated by miR-134, demonstrating the system is robust with respect to false positives. It has been previously demonstrated that in an assay of this kind, the electrocatalytic current decreases by approximately a factor of 4 in the presence of a 1 base mismatch, which further confirms this sensor is sufficiently specific^37, 42, 43^. Equally, the electrocatalytic reaction causes any non-specifically bound proteins to be desorbed from the nanoparticle surface, making the response insensitive to non-specific binding.

### Detection of miR-134 in epilepsy patient plasma and CSF

Finally, we sought to assess the performance of the electrochemical, microfluidic sensor at detecting miR-134 in human plasma and cerebrospinal fluid samples. Plasma and CSF samples were obtained from healthy volunteers and from patients with drug refractory epilepsy or in non-epileptic patients who experienced status epilepticus during in-patient care (See Table 1 for patient details). The unprocessed plasma and CSF samples were injected into the sample chamber of the microfluidic disc, where the target miRNA was injected previously (chamber (i), Fig. 1). The microfluidic device was then spun on the experimental spin stand, as previously explained. The electrocatalytic current was measured before and after the addition of hydrogen peroxide. Table 2 shows the results of the change in current of each of the patient samples, and the corresponding concentration of miR-134 present in the sample, calculated from the calibration curve described in Fig. 3. The concentrations for these samples vary from 10 mM to 10 nM for the samples taken from patients with epilepsy or who experienced status epilepticus. For the two control samples, taken from patients without a neurological condition (patient A and patient B), the current generated was acceptably low, at the limit of detection of the calibration plot. While it is unknown at this point if these concentrations are especially high or low for epilepsy patients, these results show that miR-134 is present in larger concentrations in diagnosed patients when compared to healthy volunteers. This is consistent with data from our previous study which showed elevated miR-134 in plasma in patients with epilepsy compared to controls. The origin of the elevated miR-134 we detected in plasma and CSF here is uncertain. While miR-134 was originally reported as brain-specific it has subsequently been found present in certain peripheral organs and has been linked to some cancers^44^. It is, however, most likely that the origin is brain tissue in our samples. The mechanism of transfer from tissue to biofluid is uncertain and may involve passive leak, for example due to disruption of the blood-brain barrier or transfer via controlled mechanisms such as via exosomes. This method could also be used for monitoring the miRNA levels in a patient during treatment, or for comparisons before and after seizures, etc., due to the fast sample-to-answer turnaround time of approximately 1 hour, 45 minutes. Future studies could test this device in a clinical setting to establish practical requirements such as in a neurological intensive care unit or video-EEG monitoring suite in a hospital setting. Future work would also include integration of the assay formation and measurement steps for minimal handling. This process could be fully automated by extending the electrodes beyond the outer perimeter of the plastic substrate and locating it under pushpin connectors. And, finally, it is likely that miR-134 alone will not be sufficiently selective as a biomarker of seizure disorders. For example, there is evidence that biofluid miR-134 levels are altered in patients with mild cognitive decline. While this is unlikely to be a typical patient category for which differential diagnosis using a molecular biomarker is needed (more common would be non-epileptic attack disorder or patients with syncope or other causes of loss of consciousness) it raises the likelihood that a device should be developed that can measure 3–5 miRNAs at the same time.

**Table 2:** Summary of the electrocatalytic response for each of the patient samples.

Patient	Sample	Disease	Δi (µA)	M
A	plasma	n/a	4.73	1 pM
B	plasma	n/a	10.19	10 pM
C	plasma	TLE	63.228	1 uM
D	plasma	TLE	34.76	10 nM
E	plasma	TLE	30.57	10 nM
F	CSF	TLE	38.68	100 nM
G	CSF	SE	38.69	100 nM
H	CSF	SE	75.34	10 mM

Key: CSF, cerebrospinal fluid; TLE, temporal lobe epilepsy; SE, status epilepticus; M, molarity.

## Conclusion

In conclusion, target miRNA, associated with epilepsy is detected inside a microfluidic disc by measuring the electrocatalytic reduction of peroxide at the platinum nanoparticles functionalised with probe strand nucleic acids and brought to the surface of the electrode via complementary miRNA hybridisation in a nucleic acid sandwich assay. The microfluidic disc utilised an event triggered system and a valving technology that used the arrival of a liquid at one location to prompt the release of another liquid at another, distant location on the disc by a connecting pneumatic channel. By pre-loading each of the chambers with each step of the hybridisation process, and releasing them systematically to incubate in the electrode chamber, a sandwich assay is formed on the gold electrode via complementary miRNA hybridisation. The current generated during the electrocatalytic reduction of hydrogen peroxide at the platinum nanoparticles varies linearly between a target concertation of 1 pM to 1 µM, with a LOD of 1 pM, and shows excellent selectivity discrimination towards the closest miRNA strand (miR-758) to the miR-134 target. Significantly, we were able to measure the presence and concentration of miRNA linked to epilepsy using unprocessed human plasma and human cerebrospinal fluid samples. The current generated from these patient samples were within the range of the calibration plot. A minimal current was obtained for the samples from healthy volunteers. This shows the feasibility of using this system as a detection method for miRNA.

In summary, this study demonstrates a novel electrochemical detection based on electrocatalytic platinum nanoparticles inside a centrifugal microfluidic device where the sandwich assay is formed using an event triggered release system, suitable for the rapid point-of-care detection of low abundance biomarkers of disease.
